# Association of Iron Deficiency With Acute Exacerbations of Pediatric Atopic Diseases: A Cross-Sectional Study

**DOI:** 10.7759/cureus.111803

**Published:** 2026-06-30

**Authors:** Hema Latha Gundu, Arun Kumar Dash, Amit Ranjan Rup, Jyoti Ranjan Behera, Sanjay Kumar Sahu

**Affiliations:** 1 Pediatrics, Kalinga Institute of Medical Sciences, Bhubaneswar, IND

**Keywords:** allergic rhinitis, asthma, atopic dermatitis, exacerbation, iron deficiency, tsat

## Abstract

Background: Iron deficiency and allergic diseases are significant global health problems, particularly among children. Iron deficiency may contribute to immune dysregulation, which has been implicated in the pathogenesis and exacerbation of atopic diseases. This study examined the relationship between iron deficiency, as indicated by transferrin saturation (TSAT), and acute exacerbation of allergic diseases among children.

Methods: A cross-sectional observational study was conducted over a 24-month period (March 2023 to February 2025) at the Department of Pediatrics, KIMS, Bhubaneswar. A total of 240 children aged six months to 18 years were enrolled and divided into three groups (n = 80 each): Group 1 (atopic with acute exacerbation), Group 2 (atopic without exacerbation), and Group 3 (non-atopic controls). Hematological indices and iron profile parameters, including TSAT, were measured. TSAT <16% was defined as iron deficiency. Binary logistic regression analysis was performed, and predicted probabilities of acute exacerbation at varying TSAT thresholds were estimated.

Results: Iron deficiency was highest in the exacerbation group (51.25%) compared with stable atopic children (42.5%) and controls (23.75%). Children with acute exacerbation experienced much lower mean TSAT (15.45 ± 4.47%) compared to those with no exacerbation (17.45 ± 4.18%) and controls (18.37 ± 4.68%) (p = 0.0002). Children with acute exacerbations were 3.38 times more likely to have iron deficiency than non-atopic controls (OR = 3.38; 95% CI: 1.72-6.64; p = 0.0004). Binary logistic regression identified TSAT as an important predictor of allergy flare-ups, with each 1% decrease linked to a 12% higher risk of exacerbation.

Conclusion: Iron deficiency, particularly low TSAT, is associated with acute allergic exacerbations in children. Routine TSAT screening in pediatric patients with atopic diseases may help identify children at increased risk of exacerbations and facilitate early intervention.

## Introduction

Allergic diseases represent a wide spectrum of disorders that occur due to hypersensitivity reactions to usually benign environmental agents and have varied presentations that may change their intensity over time [1]. The most common chronic atopic diseases in children include asthma, atopic dermatitis (AD), allergic rhinitis, and food allergy, which can lead to severe morbidity and, in some cases, death [1]. It is estimated that about 40-50% of school-going children around the world are sensitive to one or more common allergens [2]. The notable increase in the prevalence of atopy in recent decades is attributed to environmental determinants such as tobacco smoke exposure, indoor and outdoor allergens, respiratory viruses, obesity, and changing lifestyle patterns [1]. Studies are still being conducted to determine the possible risk factors related to atopic diseases and their rate of exacerbations [1]. Iron deficiency and allergic diseases are serious health issues, especially in children across the globe. About 30% of the global population suffers from iron deficiency [3]. Children with iron deficiency may experience immune dysregulation because iron is an essential micronutrient involved in the regulation of both innate and adaptive immune responses. Iron deficiency can disturb immune balance by impairing regulatory T-cell function and promoting a Th2-dominant response, which is central to the development of atopic diseases. This shift may enhance the production of IL-4, IL-5, and IL-13, increase IgE synthesis, and stimulate mast cell and eosinophil activity, thereby exacerbating atopic diseases [4]. An association between iron deficiency and the severity and progression of allergic diseases has been reported, whereby lower iron levels are associated with more frequent and severe exacerbations in atopic children [5]. To prevent and manage allergic diseases, it is necessary to identify modifiable factors, such as micronutrient adequacy, dietary patterns, and allergen exposure [6]. Serum ferritin may be elevated during inflammation, reducing its usefulness as a standalone marker of iron deficiency. Therefore, transferrin saturation (TSAT), which reflects available iron, is commonly used, with levels below 16% indicating iron deficiency in children [7]. The association between TSAT and the activity of allergic diseases in pediatric populations is poorly studied. Therefore, this study aimed to evaluate the association between iron deficiency, as reflected by low TSAT, and acute exacerbations of atopic diseases in children.

## Materials and methods

This cross-sectional observational study was conducted from March 2023 to February 2025 in the pediatric outpatient and inpatient departments in the Department of Pediatrics at Kalinga Institute of Medical Sciences, after obtaining written informed consent from parents or legal guardians. The study was conducted following approval from the Institutional Research Committee of KIMS, Bhubaneswar (KIMS/SLRC/2023/100, dated February 14, 2023). Ethical clearance was obtained from the Institutional Ethics Committee (IEC) of KIMS (Approval No. KIIT/KIMS/IEC/1265/2023), and the study adhered to the principles outlined in the Declaration of Helsinki [8]. The study included children aged six months to 18 years with newly diagnosed or previously established allergic rhinitis, asthma, or AD, along with age- and sex-matched healthy controls. Allergic rhinitis was diagnosed according to the 2017 Allergic Rhinitis and Its Impact on Asthma (ARIA) guidelines in children aged ≥2 years. Asthma was diagnosed based on the 2023 Global Initiative for Asthma (GINA) guidelines, including specific recommendations for children under five years of age. AD was diagnosed using the Hanifin and Rajka criteria [9-11]. Acute exacerbation was defined as a Total Nasal Symptom Score (TNSS) ≥8 for allergic rhinitis, a Pediatric Respiratory Assessment Measure (PRAM) score ≥4 for asthma, and a SCORing Atopic Dermatitis (SCORAD) >50 for AD [9,12,13].

Healthy controls were recruited from children attending the same hospital for non-allergic, non-inflammatory conditions, such as minor injuries, functional abdominal pain, or routine health check-ups, and were frequency-matched to the atopic groups by age (6 months-5 years, 6-11 years, and 12-18 years) and sex.

**Figure 1:**
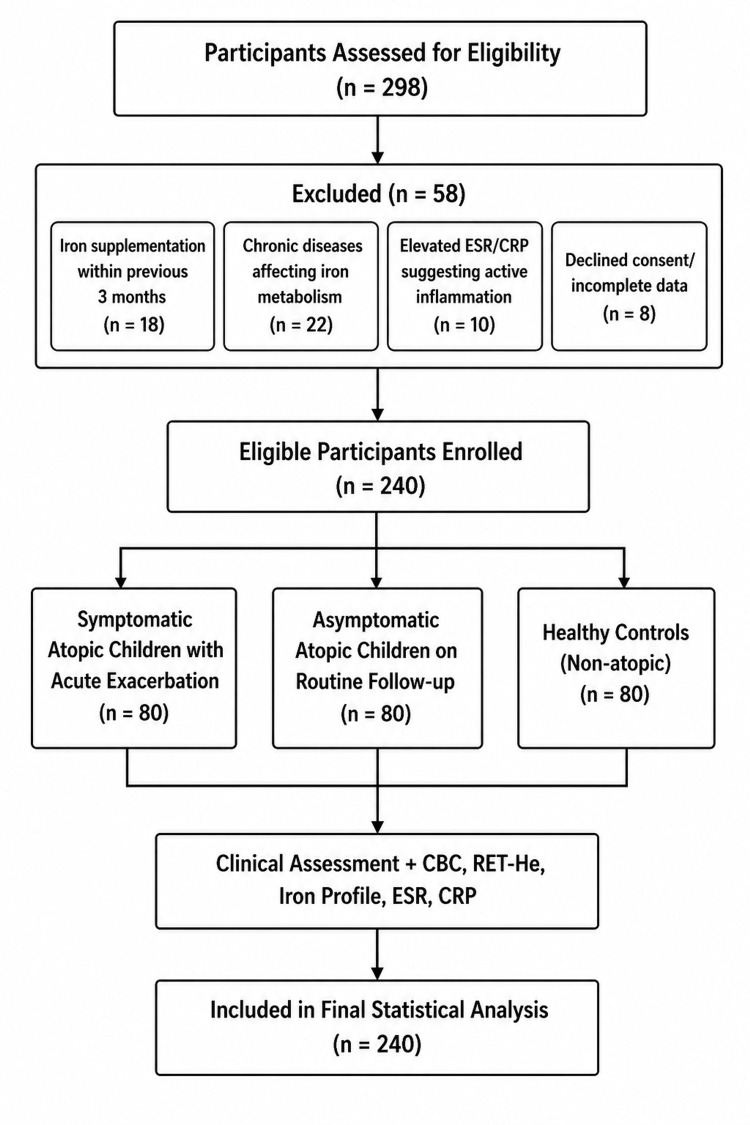
Participant flow diagram of the study CBC: complete blood count, RET-He: reticulocyte hemoglobin content, CRP: C-reactive protein, ESR: erythrocyte sedimentation rate

Children receiving iron supplementation or who had received iron therapy within the preceding three months were excluded. Additional exclusion criteria included chronic systemic diseases known to affect iron metabolism, such as chronic kidney disease, chronic liver disease, congenital heart disease, malignancy, hemoglobinopathies, autoimmune disorders, inflammatory bowel disease, tuberculosis, and other chronic inflammatory conditions. Children with elevated inflammatory markers, defined as ESR above age-specific reference ranges or C-reactive protein (CRP) >10 mg/L, were also excluded [14]. The flow of participants through the study, including eligibility assessment, exclusions, enrollment, group allocation, and final inclusion in the analysis, is presented in Figure 1.

Sample size was calculated using the formula for comparison of two independent proportions: n = [(Zα/2 + Zβ)2 × {P1(1-P1) + P2(1-P2)}] / (P1-P2)2, where Zα/2 = 1.96 for a 95% CI, Zβ = 0.84 for 80% power, P1 = prevalence of iron deficiency in the exacerbation group = 52%, and P2 = prevalence in the controls = 28% [15]. Consecutive eligible participants were recruited until the required sample size was achieved. The calculated sample size was 62 participants per group. Allowing for potential incomplete data, a minimum of 68 participants were required; however, 80 participants were enrolled in each group to improve statistical precision.

The study participants were divided into three groups: symptomatic atopic children with acute exacerbations, asymptomatic atopic children on routine follow-up, and non-atopic children with unrelated non-allergic illnesses as controls.

A structured proforma was used to collect a detailed history, relevant demographic information, vital signs, and laboratory investigations of all eligible participants. The laboratory tests included an extended complete blood count with hemoglobin (Hb), RBC count, mean corpuscular volume (MCV), mean corpuscular hemoglobin (MCH), mean corpuscular hemoglobin concentration (MCHC), packed cell volume (PCV), platelet count, reticulocyte count, red cell distribution width (RDW), and reticulocyte hemoglobin content (RET-He). Iron profile analysis included measurements of serum iron, TSAT, serum ferritin, total iron-binding capacity (TIBC) and unsaturated iron-binding capacity (UIBC). ESR and CRP were done to rule out inflammatory conditions. Complete blood count analysis was performed using a Sysmex XN-3100 automated hematology analyzer (Sysmex Corporation, Kobe, Japan), with an analysis time of approximately 30 seconds per sample. The Vitros 5600 integrated analyzer (Ortho Clinical Diagnostics, Raritan, New Jersey) was used to measure iron profile parameters. Iron deficiency was defined as a TSAT of less than 16%, in accordance with the population-based reference ranges reported by the Canadian Laboratory Initiative on Pediatric Reference Intervals (CALIPER) dataset and standard pediatric hematology guidelines [7,16].

The data were entered into a Microsoft Excel (Microsoft Office, Redmond, Washington) spreadsheet and analyzed with IBM SPSS Statistics for Windows, Version 25 (Released 2017; IBM Corp., Armonk, New York) [17]. Categorical variables were represented by frequencies and percentages and were analyzed using the chi-square test or Fisher's exact test, as necessary. The normality of continuous variables was assessed using the Shapiro-Wilk test. Continuous variables were presented as mean ± standard deviation (SD) or median interquartile range (IQR), according to their distribution. One-way ANOVA was used to make inter-group comparisons with post-hoc Tukey correction when the data were normally distributed, and the Kruskal-Wallis test was used with Dunn's post-hoc correction when the data were non-parametric. To further evaluate the association between TSAT and acute allergic exacerbations, binary logistic regression analysis was performed. Acute exacerbation status (yes/no) was considered the dependent variable, while TSAT (%) was entered as the independent predictor. Regression coefficients, odds ratios (ORs), 95% confidence intervals (CIs), and p-values were calculated. The model generated the probability of exacerbation across the observed range of TSAT values, allowing assessment of how exacerbation risk changed with iron status. A p-value < 0.05 was considered statistically significant.

## Results

A total of 240 children were enrolled. The participants were equally divided into three groups: children with acute allergic exacerbations (n = 80), children with stable atopic disease (n = 80), and non-atopic controls (n = 80). The 6-11-year age group was the most represented across all cohorts. The median ages were seven years (IQR: 3-11) in the exacerbation group, nine years (IQR: 4.5-12) in the no-exacerbation group, and eight years (IQR: 4.5-12) among controls, while male participants constituted 46 (57.5%), 44 (55%), and 43 (53.7%) children, respectively, with no statistically significant differences in age or sex distribution across the groups.

Among children with acute allergic exacerbations, asthma was the most common diagnosis, affecting 39 (48.75%), followed by AD in 23 (28.75%) and allergic rhinitis in 18 (22.5%) children. Asthma and allergic rhinitis were more common among males, whereas skin manifestations showed a balanced sex distribution. Mean Hb levels were significantly lower in the exacerbation group (11.29 ± 1.14 g/dL) compared with the no-exacerbation group (11.57 ± 1.30 g/dL) and controls (11.87 ± 1.20 g/dL) (p = 0.01). MCV was lowest in the exacerbation group (76.30 ± 6.10 fL) compared with the no-exacerbation (77.90 ± 5.04 fL) and control groups (79.61 ± 5.52 fL), showing statistically significant intergroup variation (p = 0.001). MCH also differed significantly across groups (25.18 ± 2.44 pg vs. 26.18 ± 2.12 pg vs. 26.65 ± 2.43 pg; p = 0.0004). Platelet counts were significantly higher in both atopic groups as compared to controls (p = 0.007). RET-He, reticulocyte count, and RDW did not demonstrate statistically significant differences among groups (p > 0.05). Patients with exacerbations exhibited a significant pattern of reduced iron stores and increased iron-binding capacity compared to non-exacerbation and control groups, as depicted in Table 1.

**Table 1:** Comparison of iron profile parameters across all the study groups. IQR: interquartile range; TSAT: transferrin saturation; TIBC: total iron-binding capacity; UIBC: unsaturated iron-binding capacity.

Parameter	Exacerbation median (IQR)	No exacerbation median (IQR)	Controls median (IQR)	p-value
Serum iron (µg/dL)	42.7 (34.00-53.95)	48.10 (41.92-59.32)	53.00 (42.88-64.08)	<0.001
TIBC (µg/dL)	379.25 (350.50-403.13)	348.05 (322.22-381.17)	349.70 (325.33-372.60)	0.0007
UIBC (µg/dL)	327.00 (304.08-353.55)	309.05 (286.48-329.90)	297.95 (273.88-329.33)	<0.001
TSAT (%)	15.85 (13.15-19.00)	17.65 (14.20-20.23)	18.35 (16.18-21.02)	<0.001

Serum ferritin levels were lowest in the exacerbation group (33.39 ng/mL; IQR: 28.43-43.08), followed by the no-exacerbation group (37.27 ng/mL; IQR: 27.85-49.25) and controls (43.98 ng/mL; IQR: 28.94-57.01), with a statistically significant difference among the groups (p = 0.04). The prevalence of iron deficiency (TSAT < 16%) was highest in the exacerbation group (41/80; 51.25%), followed by the no-exacerbation group (34/80; 42.5%) and controls (19/80; 23.75%). Children with acute exacerbations had significantly higher odds of iron deficiency compared with non-atopic controls (OR = 3.38; 95% CI: 1.72-6.64; p = 0.0004). The likelihood of iron deficiency was also significantly higher in the no exacerbation group than in the control group (OR = 2.37; 95% CI: 1.20-4.68; p = 0.01). However, the difference between the two atopic groups was not statistically significant (OR = 1.42; 95% CI: 0.76-2.65; p = 0.27). Pairwise comparison analysis (Table 2) demonstrated significantly lower TSAT levels in the exacerbation group compared to both the control and no-exacerbation groups. However, no significant difference was observed between the no-exacerbation group and the control group.

**Table 2:** Pairwise comparison of transferrin saturation (TSAT) values across all the study groups. CI: confidence interval.

Comparison	Mean difference (%)	p-value	95% CI lower	95% CI upper
Controls vs. exacerbation	−2.93	0.0001	−4.58	−1.27
Controls vs. no exacerbation	−0.92	0.39	−2.58	0.74
Exacerbation vs. no exacerbation	2.00	0.01	0.35	3.66

**Figure 2:**
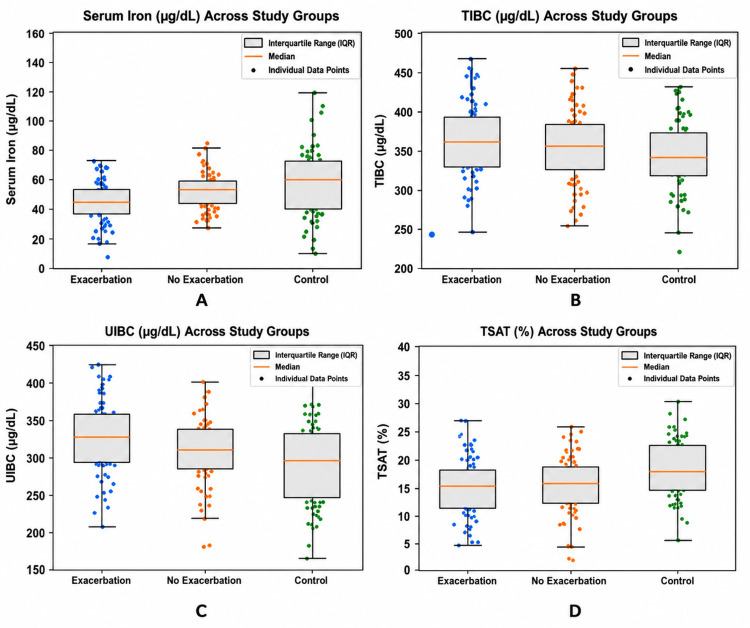
Box-whisker and jitter plots for the data shown in Tables 1 and 2. A. Serum iron distribution across study groups. B. Total iron-binding capacity (TIBC) distribution across study groups. C. Unsaturated iron-binding capacity (UIBC) distribution across study groups. D. Transferrin saturation (TSAT) distribution across study groups. The central horizontal line within each box represents the median. The box denotes the interquartile range (IQR), whiskers indicate variability, and scattered points represent individual simulated observations.

Figure 2 shows box-whisker and jitter plots for the data shown in Tables 1 and 2.

To further characterize the association between iron status and exacerbation risk, TSAT was evaluated both as a continuous variable using binary logistic regression and as a categorical variable using a clinically relevant cutoff of <16% in a contingency table analysis.

**Figure 3:**
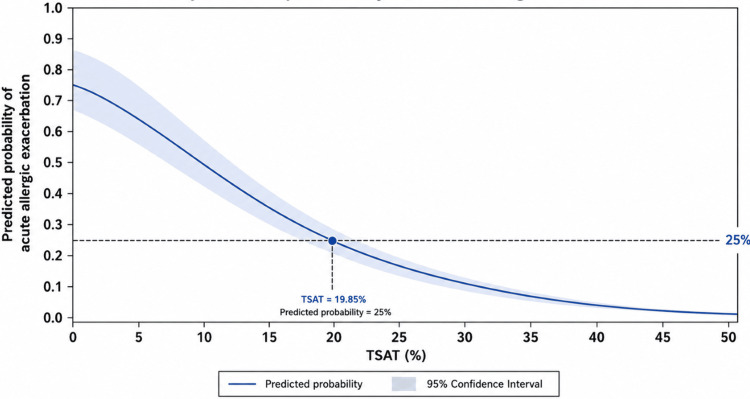
Logistic regression: relationship between TSAT and predicted probability of acute allergic exacerbation. TSAT: transferrin saturation.

Binary logistic regression analysis (Figure 3) demonstrated a significant inverse relationship between TSAT and acute allergic exacerbation. Each 1% increase in TSAT was associated with an approximately 12% reduction in the odds of exacerbation (OR = 0.88; 95% CI: 0.83-0.94; p < 0.001). The model further showed that the probability of acute allergic exacerbation increased progressively as TSAT levels declined, suggesting that lower iron availability is associated with a greater risk of exacerbation. This pattern mirrors the findings in Table 1, where median TSAT levels were lowest in children with acute exacerbations and highest in healthy controls, indicating that lower TSAT is associated with a higher risk of allergic flare-ups.

As shown in Table 3, contingency analysis based on the predefined TSAT threshold of 16% demonstrated a significant association between iron deficiency and acute allergic exacerbation, with children having TSAT < 16% exhibiting more than twofold higher odds of exacerbation compared with those having TSAT ≥ 16% (OR = 2.12; 95% CI: 1.23-3.66; p = 0.006).

**Table 3:** Contingency table showing the association between iron deficiency and acute allergic exacerbation. TSAT: transferrin saturation; CI: confidence interval.

TSAT status	Acute exacerbation (n = 80)	No acute exacerbation (n = 160)	Odds ratio (95% CI)	p-value
TSAT < 16%	41 (51.3%)	53 (33.1%)	2.12 (1.24-3.64)	0.006
TSAT ≥ 16%	39 (48.7%)	107 (66.9%)		

## Discussion

The current study aims to determine TSAT systematically in different stages of clinical atopic disease in children, such as acute exacerbation, stable atopy, and non-atopic controls, using a comparative cross-sectional study design.

Our cohort, which primarily comprises school-aged children (6-11 years), is consistent with published literature. Ayuk et al. also reported that two-thirds of their study population were children aged 4-11 years, with a median age of 10 years [18]. The United States Centers for Disease Control and Prevention (CDC, 2021) data proved that the prevalence of seasonal allergies was higher in children aged 6-11 years compared to younger children [19]. This epidemiological trend could be due to similar environments with increased allergen load, increased outdoor activities, recurring respiratory exposures at school, and an immune system undergoing a gradual Th2 shift in response to school-related antigen exposures [20]. Conversely, the Global Burden of Disease 2019 study identified children younger than five years as the age group with the highest prevalence of asthma and AD [21], implying that environmental exposures and the changing immune maturation process could be changing the temporal epidemiological patterns.

There was a slight male predominance in all groups, which aligns with the global trends that show greater prevalence of allergic diseases in boys during childhood, with a shift to female preponderance in adolescence and adulthood [22]. This sex difference may be caused by hormonal and developmental factors, such as prepubertal uninhibited stimulation of pro-allergic Th2 pathways and delayed maturation of regulatory T-cells in boys [23]. The International Study of Asthma and Allergies in Childhood (ISAAC) found a similar male preponderance in airway hyperreactivity among younger children [24]. Interestingly, in our 12-18-year subgroup in the stable atopy group, a greater female-to-male ratio was noted, which is a reflection of the anticipated pubertal hormonal change.

Significantly lower hemoglobin levels were observed in the exacerbation group, supporting the findings of Ha et al., who linked reduced hemoglobin to impaired pulmonary function in asthmatic children, and Elbahy et al., who identified low hemoglobin as an independent risk factor for asthma [15,25]. This decrease in hemoglobin can be partially due to inhibition of erythropoietin by pro-inflammatory cytokines, indicating anemia of chronic disease overlaid on functional iron deficiency. The exacerbation group has significantly lower MCV and MCH values, which are parallel with the results of Ayuk et al. and Rhew et al. [18,26]. Children with acute exacerbations had lower serum iron and ferritin levels than stable and control groups, consistent with previous studies reporting reduced iron and ferritin in asthmatic and allergic children [25,27]. Notably, the numerous outliers found in serum ferritin values in our study may be explained by the fact that ferritin is an acute-phase reactant, which can be increased in the case of active inflammation, thus restricting its application as a single diagnostic marker for iron deficiency during allergic flare-ups [28].

The primary finding of this study is that iron deficiency (TSAT < 16%) is significantly associated with acute allergic exacerbation. These values are consistent with those of Serbs et al., who found a much higher prevalence of iron deficiency anemia (IDA) in children with AD than in non-atopic controls, and with the large-scale study by Rhew and Oh [26,29]. Notably, Li et al. conducted the Mendelian randomization analysis and discovered that genetically predicted IDA was causally linked to 37% higher asthma risk (OR = 1.37; 95% CI: 1.09-1.72), which was stronger causal evidence as compared to observational studies [30]. In our logistic regression model, TSAT was statistically significant as an independent predictor of acute allergic exacerbation, and each 1% decrease in TSAT was associated with 12.6% higher odds of exacerbation. This mechanistic interaction is supported by cytokine-induced hepcidin expression in allergic inflammation, which inhibits the duodenal uptake of iron and traps iron in macrophages and hepatocytes, thereby depleting bioavailable transferrin-bound iron [31]. TSAT levels were significantly reduced during acute exacerbations compared with stable atopy. In contrast, no significant difference was observed between the stable atopy and control groups. This finding suggests that changes in TSAT may reflect acute inflammation-related disturbances in iron metabolism rather than a persistent underlying nutritional deficiency. Roth-Walter established that iron deficiency enhances preferential Th2-lymphocyte maintenance, IgE class switching, and degranulation of mast cells, which are the direct drivers of atopic exacerbations [5]. Roth-Walter et al. also revealed that common allergens like lipocalins can actively deprive immune cells of iron, which exacerbates sensitization [32]. All these mechanisms contribute to making TSAT not only a nutritional marker but also a dynamic measure of disease activity in atopic children. Moreover, in a cohort of 846,718 children, Rhew and Oh reported increased odds of IDA with asthma, AD, and allergic rhinitis (OR = 1.71, 1.42, and 1.25) [25]. Additionally, the logistic regression model demonstrated a graded increase in the probability of acute exacerbation as TSAT levels decreased, supporting the observed lower TSAT values among children experiencing acute allergic exacerbations.

Strengths and limitations

The key strength of this study is its three-group parallel design, allowing assessment of iron metabolism across different stages of atopy, supported by comprehensive iron and hematological profiling, strict diagnostic criteria, exclusion of recent iron supplementation, and predictive analysis using logistic regression. The cross-sectional nature of the study limits the ability to establish causal relationships, allowing only the identification of associations. Additionally, recruitment from a single tertiary care center may limit the generalizability of the findings because patients with more severe disease may be over-represented.

## Conclusions

This cross-sectional study demonstrates that iron deficiency, which is defined by low TSAT (<16%), is significantly associated with acute exacerbations of allergic diseases in children aged six months to 18 years. Children with acute allergic exacerbations were found to have much lower TSAT levels and more iron deficiency than children with stable atopy and non-atopic controls. Future prospective studies are needed to determine this relationship and assess the potential clinical value of TSAT measurement.
